# Study on the Fire Damage Characteristics of the New Qidaoliang Highway Tunnel: Field Investigation with Computational Fluid Dynamics (CFD) Back Analysis

**DOI:** 10.3390/ijerph13101014

**Published:** 2016-10-15

**Authors:** Hongpeng Lai, Shuyong Wang, Yongli Xie

**Affiliations:** 1 School of Highway, Chang’an University, Xi’an 710000, China; xieyl@263.net; 2 Fujian Communications Planning and Design Institute, Fuzhou 350000, China; wsy0618@163.com

**Keywords:** tunnel engineering, fire, damage characteristics, numerical simulation, CFD

## Abstract

In the New Qidaoliang Tunnel (China), a rear-end collision of two tanker trunks caused a fire. To understand the damage characteristics of the tunnel lining structure, in situ investigation was performed. The results show that the fire in the tunnel induced spallation of tunnel lining concrete covering 856 m^3^; the length of road surface damage reached 650 m; the sectional area had a maximum 4% increase, and the mechanical and electrical facilities were severely damaged. The maximum area loss happened at the fire spot with maximum observed concrete spallation up to a thickness of 35.4 cm. The strength of vault and side wall concrete near the fire source was significantly reduced. The loss of concrete strength of the side wall near the inner surface of tunnel was larger than that near the surrounding rock. In order to perform back analysis of the effect of thermal load on lining structure, simplified numerical simulation using computational fluid dynamics (CFD) was also performed, repeating the fire scenario. The simulated results showed that from the fire breaking out to the point of becoming steady, the tunnel experienced processes of small-scale warming, swirl around fire, backflow, and longitudinal turbulent flow. The influence range of the tunnel internal temperature on the longitudinal downstream was far greater than on the upstream, while the high temperature upstream and downstream of the transverse fire source mainly centered on the vault or the higher vault waist. The temperature of each part of the tunnel near the fire source had no obvious stratification phenomenon. The temperature of the vault lining upstream and downstream near the fire source was the highest. The numerical simulation is found to be in good agreement with the field observations.

## 1. Introduction

Uncontrolled fires happening in road tunnels in confined spaces can cause catastrophic consequences. It is reported that an outbreak of fire caused by collisions of multiple heavy vehicles and passenger cars in the Mont Blanc tunnel (France/Italy) in 1999 caused 39 deaths and left 30 people injured; a fire caused by car collisions in Hongjimun tunnel (Korea) in 2003 left more than 40 people injured [1]. Fire in road tunnels not only poses serious threats to people, but also severely damages the tunnel structures. Tunnel linings were found to be spalled up to a depth of 400 mm in some cases and the strength of reinforced concrete was significantly reduced after fire [2]. Post-fire structural repair can induce tunnel closure causing significant economic impact. According to incomplete statistics around the world, serious road tunnel fires have caused casualties and significant property loss [1].

Increasing frequency of fire incidents in tunnels has led to greater research efforts in order to investigate fires in tunnels so that proper strategies can be adopted in the design and maintenance of tunnels against fires. Many methods have been used to study fire development in tunnels, including reduced- and/or full-scale fire experiments, numerical simulation, and analytical methods. The purpose of fire experiments is to obtain knowledge on fire development with respect to different factors [1,2,3,4,5,6,7]. For example, Oka and Atkinson [3] performed a reduced-scale fire experiment to study the specification of the longitudinal ventilation necessary to prevent upstream movement of combustion products in a tunnel fire. In order to obtain heat release rates (HRR) in different Heavy Goods Vehicle (HGV)-trailer cargos in a tunnel with forced longitudinal ventilation, Ingason and Lönnermark [4] carried out four large-scale tests in a road tunnel. The results show that peak HRRs were found in the range of 66–202 MW and the time to obtain a peak HRR was found to be in the range of 7–18.5 min from ignition. Minehiro et al. [7] performed tunnel fire experiments using a large-scale model tunnel to produce completely turbulent flow in the tunnel and investigated the backlayering distance of thermal fumes. The use of computational fluid dynamics (CFD) for fire safety design of tunnels has become increasingly popular in the past few years. Two CFD software tools, known as Fluent (ANSYS, Canonsburg, PA, USA) and Fire Dynamic Simulator (FDS) (National Institute of Standard and Technology, Gaithersburg, MD, USA), are frequently used in tunnel fire simulation [8,9,10,11,12,13,14,15,16]. For instance, Hwang and Edwards [10] used FDS to simulate floor-level fires in a ventilated tunnel and the simulation was verified by checking the computed velocity profile against experimental data. Their results show that the FDS code was capable of predicting the critical ventilation velocity in channels of various size and configuration. Tilley et al. [14] performed numerical simulations to repeat small-scale experiments of fire in an atrium and a tunnel using FDS and concluded that “the prediction of the quasi steady-state smoke region by CFD is good, especially when the experiments are well documented”. Gao and Zhang [15] pointed out that unverified CFD simulation performed by inexperienced users carries risks, because the commercial package may simplify the simulation task. Furthermore, they pointed out that analytical solution may be a better alternative for verification since it is based on the actual physical process. Gao and Zhang [15] developed an analytical solution for tunnel fire under longitudinal ventilation, and the results were compared with simulation using Fluent and FDS and verified by checking with documented experimental data. They found that the CFD simulation with FDS program for a narrow tunnel failed to conform to the same trend as that in the theory and experimental data.

It can be seen that scholars conduct much research on the tunnel fire through lots of fire tests, numerical simulation, and analytical analysis. Many positive findings have been found which greatly help to improve fire safety in tunnels. However, most of the research mainly focuses on predicting smoke spread, ventilation efficiency and heat release rate. Only a few studies focus on the effect of fire on the tunnel lining structure [2,17,18]. The current paper intends to investigate the effect of fire on the tunnel structure using detailed analysis of the characteristics of a tunnel′s lining structure after fire. In order to perform back analysis of the effect of thermal load on lining structure, simplified numerical simulation using CFD was also performed for repeating the fire scenario.

## 2. General Situation of the Tunnel and Fire

**Figure 1:**
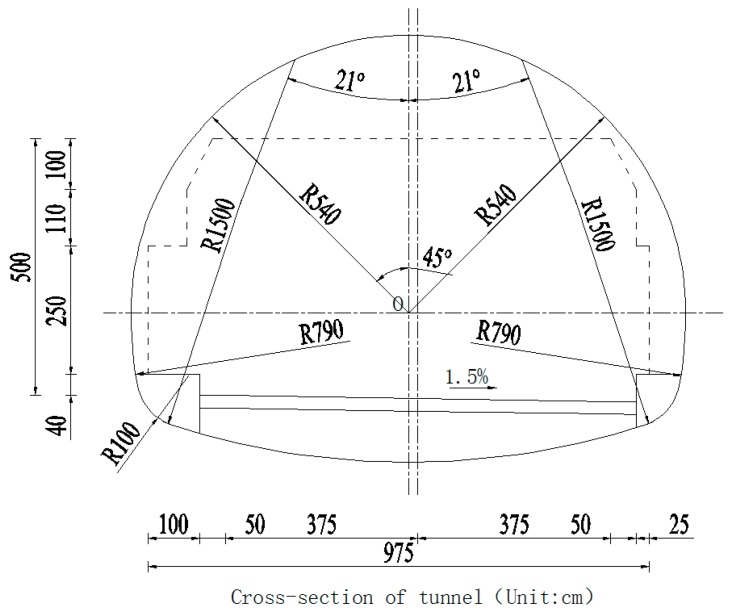
Cross-section of Qiaodaoliang Tunnel.

The New Qidaoliang Tunnel on No. 212 national highway (Lanzho–Lintao Highway) is a lengthy two-way highway tunnel, with 8073.19 m of the double-hole in length, of which the uplink is 4003.19 m, and the downlink is 4070 m. The tunnel is designed to have double-hole one-way traffic, and the space of the center lines between two holes is 45 m. The designated speed is 80 km/h, and the inner contour of the tunnel is single round, with a radius of 5.40 m and a vault 7.1 m high. The carriageway is 2 × 3.75 m in width, and the marginal strip of two sides is designed to be 0.5 m in width. The maintaining roadway with 1.00 m in width is designed on the left side in the hole, with 0.25 m in width left on the right side. The clear width of the building line of the tunnel is 9.75 m, and the clear height is 5.0 m; the longitudinal slope of the uplink is +2.05%, and the longitudinal slope of the downlink is +2.11%. A drawing of tunnel′s cross-section is shown in Figure 1.

As for the tunnel ventilation, vertical shaft plus longitudinal jet flow ventilation is adopted. A ventilation shaft with 6 m in diameter is set up on both the uplink and downlink. There are 70,900-type jet fans for the uplink and 48,900-type jet fans for the downlink, with a spacing of 110 m. The road surface of the tunnel is concrete with a thickness of 25 cm. Five emergency parking strips and five cross passages are laid out for the whole tunnel. At about 3:00 a.m. on 8 April 2011, two tank trucks rear-ended and caught fire between section K18+655 and K18+705 of the right hole of the new Qidaoliang tunnel. The naked flame in the hole was put out by the firefighters after about 2 h. Four people were killed, three trucks burned out, and two tankers were seriously damaged. Due to collision, the head of one of the tankers was seriously deformed and the head cover dropped out, while the other tanker had liquid leakage with about two tons of residual liquid left at the site. Four hours after the naked flame was put out, the visibility within the scope, about 1235 m from Lanzhou side hole mouth to the site of the accident, was 80–100 m. The visibility within the scope, about 2768 m from the Lintao side hole mouth to the site of the accident, changed with the distance from this side hole. The visibility within the scope of the first 400 m was about 60 m, while the visibility beyond the scope of the first 400 m decreased until zero rapidly. The volatilized gas content of the soot in the air and the solvent oil in the wrecked trucks in this section was higher, with obvious pungent smell and sensation of asphyxia. The temperature in the wrecked trucks was still very high, and near the trucks there was an obvious burning sensation, with white smoke billowing outward.

**Figure 2:**
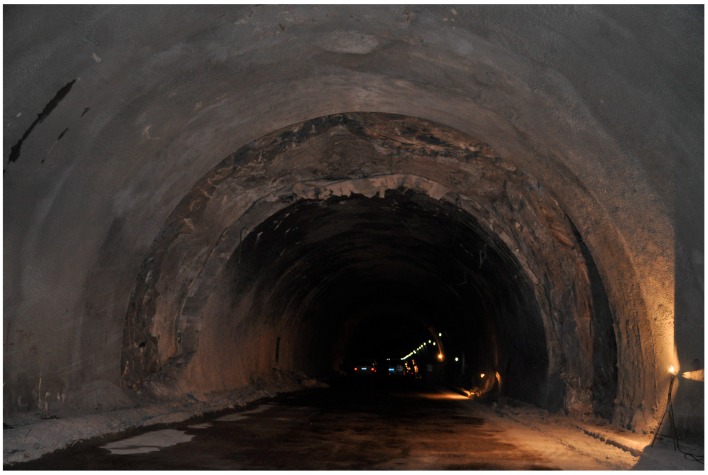
Lining of New Qidaoliang Tunnel after the fire.

Figure 2 shows the damaged tunnel lining after fire. This accident caused serious damage to lining structure, road surface, mechanical and electrical facilities, monitoring facilities, and interior finishing of the tunnel on the accident section; the large-area concrete lining within the scope of fire was spalled; the cable cradles all dropped, and all kinds of mechanical and electrical facilities were all damaged.

## 3. Analysis of Characteristics of Tunnel after Fire

### 3.1. Tunnel Dregs Condition

Due to high temperature in the tunnel, uneven complex stress was produced in the lining concrete, making large-area lining concrete crack and spall which became serious because of the cooling effect of the water used to fight the fire, and a mass of concrete debris accumulated on the road surface. It is estimated that the volume of the concrete debris was about 856 m^3^.

### 3.2. Evaluation of Strength of Tunnel Lining

#### 3.2.1. Evaluation of the Vault Strength of Lining

**Figure 3:**
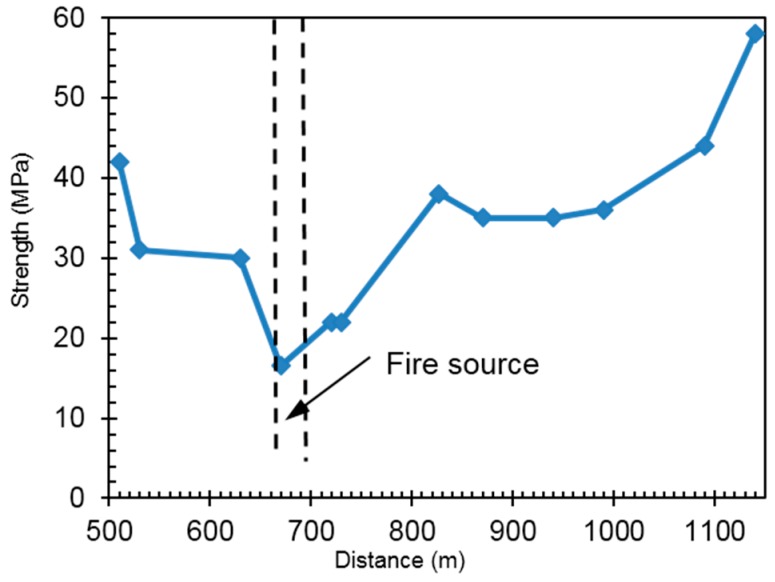
Longitudinal distribution diagram of vault concrete strength along the tunnel.

For the damaged section of tunnel, the rebound method was adopted to evaluate the concrete strength of the vault lining structure. The vault concrete strength along longitudinal direction near the fire spot is shown in Figure 3. The strength of residual lining near the fire spot decreases significantly to a minimum value of 16.5 MPa. The thermal load carried by the smoke creates an influence zone about 400 m away from the fire source, in which the strength of vault concrete decreases to some extent.

#### 3.2.2. Evaluation of the Side Wall Strength

**Figure 4:**
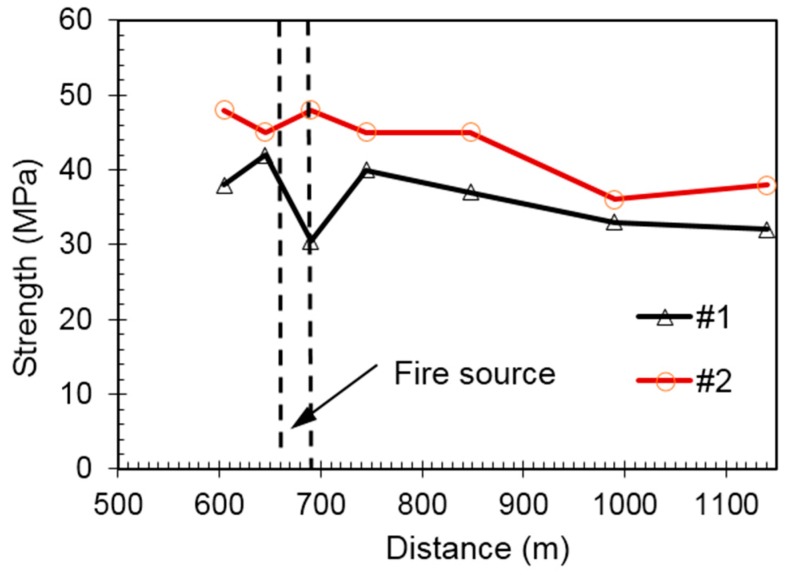
Comparison of compressive strength of #1 and #2 specimens at varied side wall locations.

For tunnel sections between K18+590 and K19+240, borehole coring method was used to collect concrete samples on the right wall of post-fire tunnel. One sample was collected every 50 m, and in total 12 samples were collected. One to two specimens were produced according the actual length of each core sample, and in total 19 cylinder specimens with dimensions of 10 cm × 10 cm were made and tested. At the same boring spot, the specimen near the inner surface of tunnel wall was marked as #1 and the specimen away from the tunnel surface closing to the surrounding rock was marked as #2. Figure 4 presents the comparison of compressive strength of #1 and #2 specimens at varied side wall locations. It is clearly shown that at the same sampling point the strength of #1 specimen is lower than that of #2, indicating that the influence of high temperature on the concrete surface is greater than that on the interior.

### 3.3. Damage Condition of Road Surface and Waterproof Board

**Figure 5:**
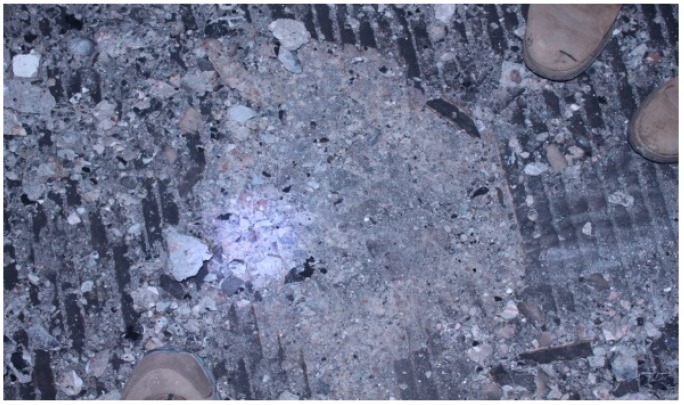
Ablation condition of surface concrete.

**Figure 6:**
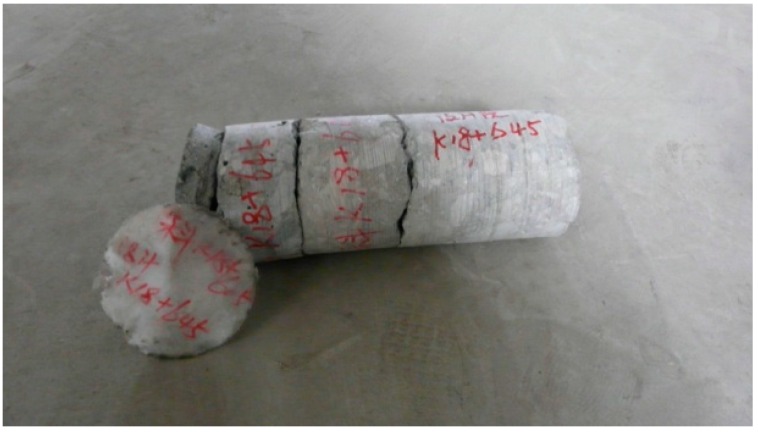
Core sample and waterproof board of right wall.

The concrete on the road surface was severely spalled by the fire, as seen in Figure 5. It is estimated that the fire has created a damaged zone about 650 m long. Boring samples taken at main fire spots (K18+645~K18+690), with a depth of up to 58 cm, show that the waterproof board is still in good condition (see Figure 6).

### 3.4. Loss of Cross-Section Area

**Figure 7:**
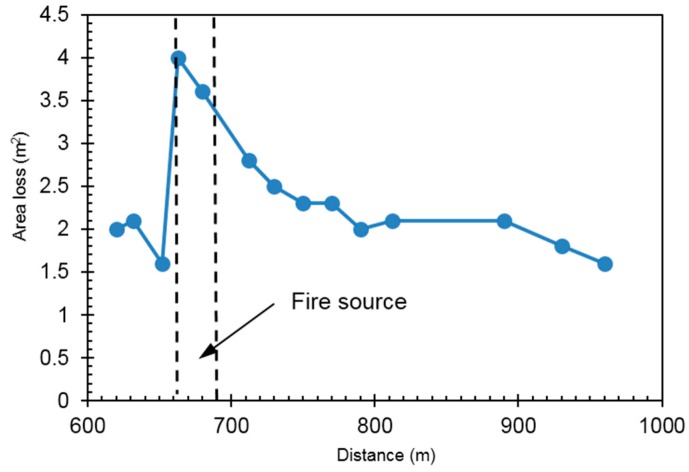
Area loss along longitudinal direction of the tunnel after fire.

Tunnel special-purpose profiler was used to determine the cross-section area after fire; in total 14 sections were measured. The difference between the measured area and the theoretical area (area loss) for varied sections were calculated and plotted in Figure 7. The maximum area loss happens at the fire spot with maximum observed concrete spallation up to a thickness of 35.4 cm.

### 3.5. Affiliated Facilities Damage Condition

**Figure 8:**
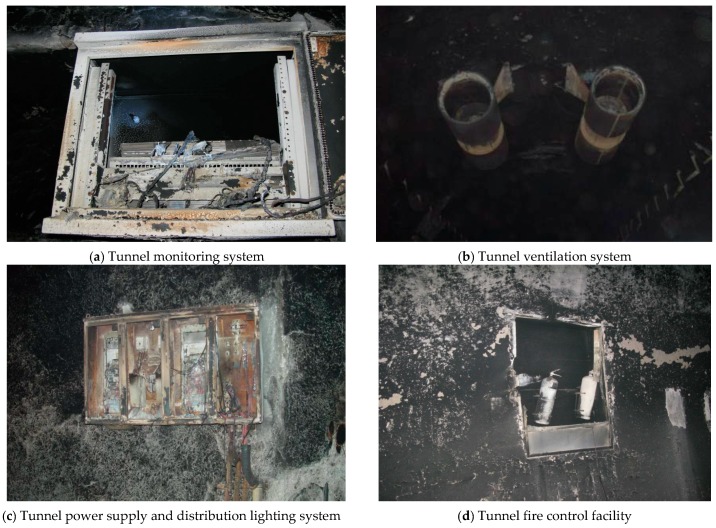
Damage to the affiliated facilities in the tunnel.

According the site inspection, the cable trough between sections K18+620 and K18+827 was seriously damaged. The cover plate of the cable trough between sections K18+450 and K19+050 needed to be changed, and the length of scattered damage on the cover plate of cable trough at other sections was estimated to be 150 m long. Mechanical and electrical facilities, including traffic mark, signs and lighting, monitor, ventilation and fire protection, were all damaged. Besides, due to the fire smoke, the spray coating of the hole wall surface become black and spalled (Figure 8).

### 3.6. Structural Damage Rating

**Figure 9:**
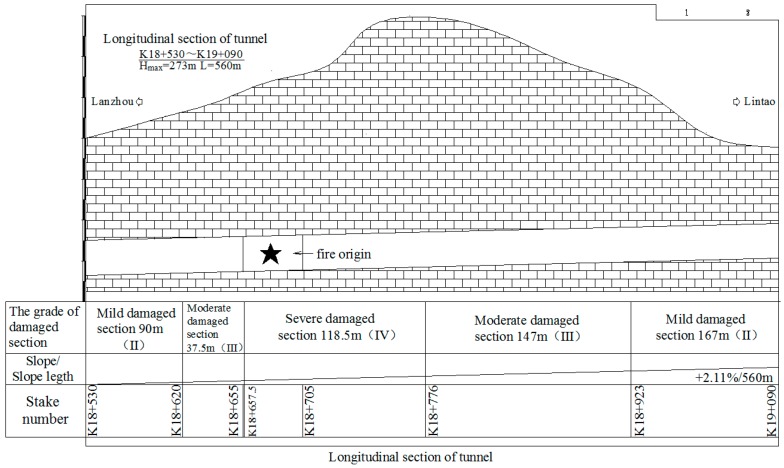
Damaged zone along the longitudinal direction of tunnel.

In accordance with the spallation lining concrete with colors from gray to pale yellow and the aluminous frit found at the scene, and on comparing the Building Structure Appraisal Standard after Fire (CECS 252:252) and materials of the fire department, it was estimated that the surface temperature of the second layer lining concrete of the tunnel vault within the main fire area was over 1000 °C. Integrating the testing analysis results of fire influence and several site surveys, the rating is provided as follows and the damaged zone along the longitudinal direction of tunnel is shown in Figure 9.

- Seriously damaged section: K18+657.5~K18+776, 118.5 m in total. A large area of the second layer lining concrete was spalled, exposing rebars in the second lining. The hole lining structure was completely damaged; the steel arch in the second lining at the draught fan was exposed, with the spalling depth of 10–35 cm. There was heavy damage to the concrete strength of the hole top, and the traffic safety facilities, mechanical and electrical facilities were all damaged. Based on the observation, the damage rating is given as grade IV.
- Moderately damaged section: K18+620~K18+657.5 and K18+776~K18+923, 184.5 m in total. The large area of the second lining concrete of this section was spalled, with spalling depth of 2–7 cm, and some parts over 10 cm. There was serious damage to the part below the hole lining and lamps, and great loss of concrete strength of the hole top. Part of the road surface, traffic safety facilities, and mechanical and electrical facilities were all damaged. Based on the observation, the damage rating is given as grade III.
- Section with minor damage: K18+530~620 and K18+923~K19+090, 257 m in total. Some areas of the second lining of concrete of this section was spalled, with spalling depth of 2–6 cm. There was less loss of concrete strength at the hole top, with the surface in good condition. The traffic safety facilities, and mechanical and electrical facilities were all damaged. Based on the observation, the damage rating is given as grade II.

## 4. Numerical Simulation of the Fire Scenario

### 4.1. Numerical Model

#### 4.1.1. Software used and Basic Assumptions

This paper uses Fluent, a typical kind of CFD software for simulating the fire scenario in order to repeat the fire incident. The basic idea of the CFD simulation is to replace the fields (such as speed field, temperature field, and concentration field, etc.) of continuous physical quantity in the space and time coordinate with the value collection of a series of limited discrete points (called node), establishing relational algebraic equation among the variable values of these discrete points according to certain principles and solving the established algebraic equation in order to obtain the appropriate solution of these variable values.

Simply put, the following assumptions and simplifications are made: the air current and temperature are uniform in the tunnel before the fire happens; adopting volume heat source model (VHS), fire source is without smoke and the hot air stands for smoke plume; the tunnel wall should be dry without any permeation; the influence of obstruction and disturbance of the car and personnel on air current is ignored; the influence of the air current generated by the ventilating system and tunnel environment on fire source is ignored, as is the influence of oxygen content on fire behavior. Although this scenario may be too simplified to simulate the real fire incident based on these assumptions, the main focus of this paper is to investigate the effect of thermal load on the tunnel lining structure.

#### 4.1.2. Tunnel Model and Boundary Conditions

##### Tunnel Model

**Figure 10:**
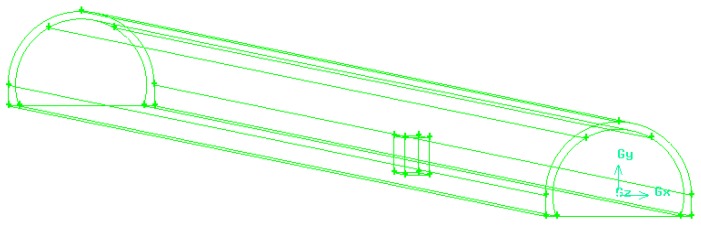
Numerical model for fire simulation.

When a fire happens in certain place in a tunnel, with the exception of smoke that can spread to areas far away, the thermal field and pressure field vary greatly only in the influence area that is close to the fire source. As the New Qidaoliang tunnel is long in length, the influence area of the fire in the longitudinal direction is large. In order to reflect the situation of the fire, we selected K18+290~K19+290 section of the New Qidaoliang tunnel (1000 m long) for numerical simulation. The K18+290 section is the tunnel inlet. Based on in situ observation, the influence of the fire on this section is very small, which could be ignored; thus, we selected this section as the control section of the wind speed of the tunnel inlet. The influence of the fire on the K19+290 section is also very small, thus, we selected this section as the pressure outlet section of the tunnel. According to the research data and design data, the fire has no influence on the tunnel lining at a depth of 62 cm; thus, we chose the lining concrete thickness of 62 cm for numerical simulation. The radius of the tunnel should be 5.4 m; vault height being 7.1 m and traffic lane width being 2 × 3.75 m with side strips which are 0.5 m wide on both sides. A maintaining roadway with a width of 1.0 m was set at the left side of the tunnel and an open space with the width of 0.25 m was set at the right side. The clear width of the construction clearance was 9.75 m, clear height being 5.0 m, and the longitudinal grade being 2.05%. The tunnel calculation model is shown in Figure 10.

##### Boundary Conditions

The tunnel inlet and outlet were set as the velocity boundary condition or pressure boundary condition. The tunnel lining was concrete material with a width of 30–100 cm. It is assumed that the outside surface of the tunnel lining is homothermal (temperature boundary) and the inner lining has one-dimensional heat conduction. As concrete is a poor conductor of heat, the actual lining thickness is generally large. The result obtained through the heat-transfer calculation is appropriate to this and the temperature variation of the outside surface of the lining is small. The initial conditions and boundary conditions of the calculation simulation are as follows: initial relative pressure in the tunnel: 0 Pa; initial air density in the tunnel: 1.225 kg/m^3^; initial temperature in the tunnel: 27 °C; initial temperature of the tunnel lining concrete: 27 °C; initial temperature of the fire source: 35 °C; relative pressure of the tunnel outlet: 0 Pa (supposing the local atmosphere is 101,325 Pa); tunnel inlet: constant velocity boundary condition; tunnel outlet: pressure outlet boundary condition; fire source: fluid source.

#### 4.1.3. Heat Release Confirmation and Combustion Model Selection

##### Heat Release Confirmation

The confirmation of the heat release rate (HHR) is crucial to numerical simulation. From the perspective of the maximum HRR of the simulated fire, present numerical simulation studies mainly focus on those below 50 MW and few studies are made on the large-scale tunnel fire simulation over 50 MW. According to the fire characteristics of the New Qidaoliang tunnel, this paper assumes the fire source as a steady state fire source and the heat release is 100 MW.

##### Combustion Model Selection

Volume heat source model (VHS) is the simplest combustion model, which leaves out the chemical reaction process. Just set a heat release rate that is equivalent to the fire source as *Q* and simulate the fire source as a heat source with a fixed volume. The fire is deemed not to spread. VHS model is simple and practicable, which simplifies calculation process, reduces calculated amount, and meets the requirement of fire risk analysis. The fire source of this calculation simulation is at K18+665~K18+685 and its size is 20 × 2 × 3 m.

#### 4.1.4. Calculation Model Selection

This paper selects the Re-Normalisation Group (RNG) k−ε model as the turbulence model, which derives from strict statistical techniques and is similar to the standard k−ε model, yet the former has the following improvements: (1) the RNG model adds a condition to ε equation, which effectively improves the accuracy; (2) the RNG model takes the turbulence vortex into consideration and improves its accuracy; (3) the RNG theory provides the turbulence Prandtl number with an analytic formula while the standard k−ε model uses constants provided by users; (4) the standard k−ε model is a kind of high Reynolds number model while the RNG theory provides an analytical formula that takes low Reynolds number flow viscosity into consideration. These characteristics make the RNG k−ε model have a higher reliability and accuracy in wider flow than the standard k−ε model.

(1) $$\rho \frac{dk}{dt}=\frac{\partial }{\partial x_{i}}[(\alpha_{k}\mu_{eff})\frac{\partial k}{\partial x_{i}}]+G_{k}+G_{b}-\rho \varepsilon -Y_{M}$$

(2) $$\rho \frac{d\varepsilon }{dt}=\frac{\partial }{\partial x_{i}}[(\alpha_{\varepsilon }\mu_{eff})\frac{\partial \varepsilon }{\partial x_{i}}]+C_{1\varepsilon }\frac{\varepsilon }{k}(G_{k}+C_{3\varepsilon }G_{b})-C_{2\varepsilon }\rho \frac{\varepsilon^{2}}{k}-R$$

In Formulas (1) and (2), $G_{k}$ stands for turbulence energy generation caused by average velocity gradient; $G_{b}$ stands for turbulence energy generation caused by buoyancy influence; $Y_{M}$ stands for the influence of compressible turbulent fluctuation swell on total dissipation rating; $\alpha_{k}$ and $\alpha_{\varepsilon }$ stand for reciprocal of the effective turbulence Prandtl number of turbulence energy *k* and dissipation rating ε respectively. For the simulation of handling low Reynolds number and near-wall flow, the calculation formula of the turbulence viscosity coefficient is $d(\frac{\rho^{2}k}{\sqrt{\varepsilon \mu }})=1.72\frac{\tilde{v}}{\sqrt{{\tilde{v}}^{3}}-1-C_{\nu }}d\tilde{v}$, v˜=μeffμ, $C_{\nu }\approx 100$. For the simulation of handling high Reynolds number, the calculation formula of the turbulence viscosity coefficient is $\mu_{t}=\rho C_{\mu }\frac{k^{2}}{\varepsilon }$, $C_{1\varepsilon }$ = 1.42, $C_{2\varepsilon }$ = 1.68.

The Discrete Ordinates (DO) model is selected as the radiation model because it can calculate all the radiation problems of the optical thickness and its calculation range covers various radiation problems such as surface radiation, translucent medium radiation, and participatory medium radiation occurs in combustion, etc.

#### 4.1.5. Input Parameters in the Simulation

The input parameters used in the simulation are selected as follows: air density in normal temperature: 1.225 kg/m^3^; gravitational acceleration: 9.81 m/s^2^; specific heat capacity of the air: 1006.43 J/(kg·K); concrete density: 2400 kg/m^3^; specific heat capacity of the concrete: 1100 J/(kg·K). According to the site research data, the time between the beginning of the fire and when the fire was extinguished in the New Qidaoliang tunnel was about 2 h. The ventilating velocity in the tunnel was 2.5 m/s when the fire occurred.

### 4.2. Calculation Result Analysis

#### 4.2.1. Longitudinal Temperature Field Contribution Rule in the Tunnel

**Figure 11:**
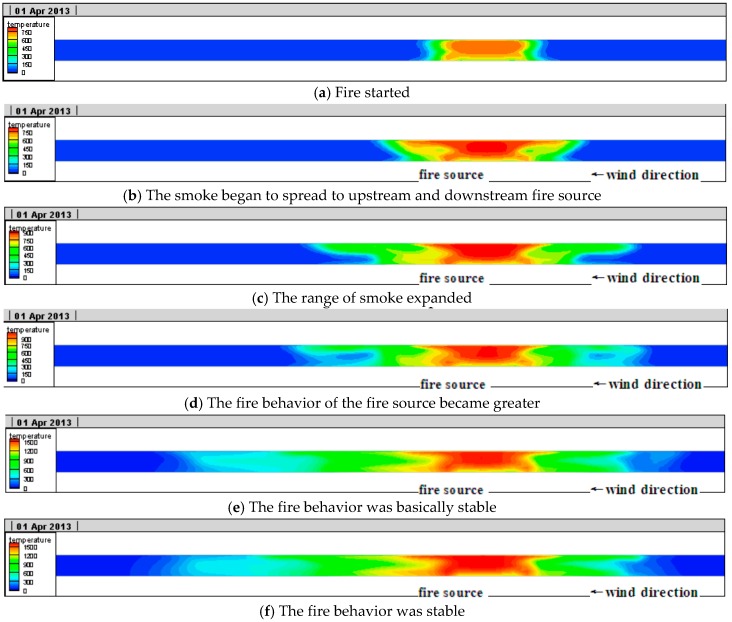
Longitudinal temperature in the tunnel.

The longitudinal temperature variations in the tunnel are as shown in Figure 11.

In Figure 11 it can be seen that when the fire started (Figure 11a), there was temperature variation only in a small area at the fire source in the tunnel. As the smoke began to spread upstream and downstream (Figure 11b) vortex appeared around the fire source. Then, the range of smoke further expanded (Figure 11c), and because of the buoyancy influence, the smoke spread faster in the tunnel vault than any other areas and backflow appeared. The behavior of the fire source became greater (Figure 11d), due to the influence of longitudinal ventilation, buoyancy, and temperature difference, turbulence appeared in the longitudinal tunnel. A small area of disturbance in smoke appeared; when the fire behavior was basically stable (Figure 11e), due to the influence of longitudinal ventilation and buoyancy, etc., the speed of the smoke spread to the upstream of the fire source clearly slowed down and the speed of the smoke spread downstream of the fire source clearly accelerated. Finally, (Figure 11f), the smoke didn’t backflow upstream any longer and the temperature spread downstream was basically stable. It can be also seen from Figure 11 that as the distance from the fire source increased in the tunnel, the heat radiation of the fire source reduced quickly and the temperature of the heat smoke airflow declined gradually due to the cooling influence of the tunnel wall. Moreover, due to the influence of longitudinal wind speed, the smoke in the tunnel gathered downstream of the fire source, which made the temperature areas in the downstream higher than areas in the upstream with the same distance from the fire. The heat release of the fire source this time was relatively large and the air with a temperature exceeding 100 °C reached 526 m in the tunnel according to the numerical calculation, which was basically in accordance with the damage situation of lining structure in situ.

#### 4.2.2. Lateral Temperature Field in the Tunnel

**Figure 12:**
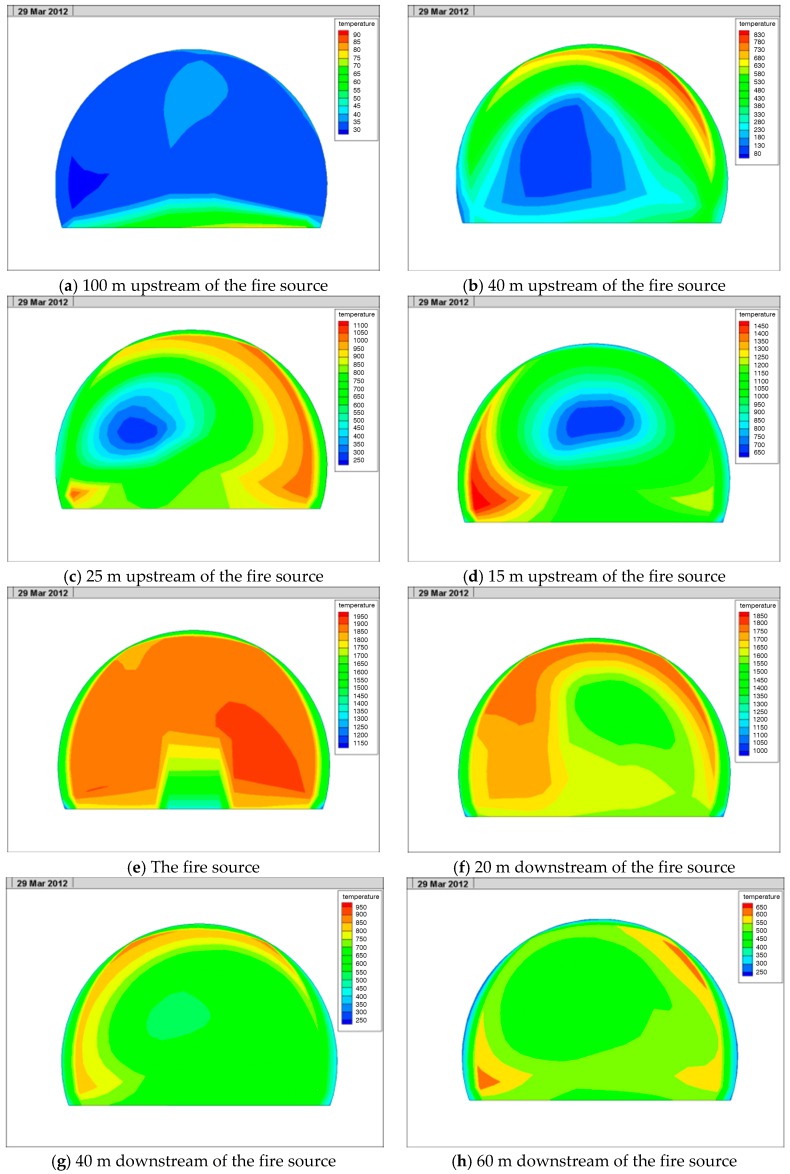

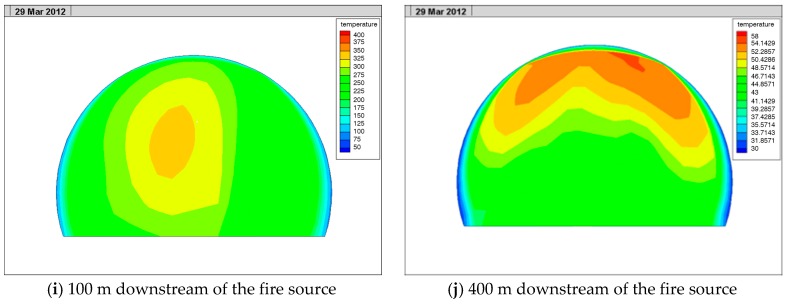
Cross section temperature in the tunnel.

The cross-section temperature distribution at different areas in the tunnel is shown in Figure 12.

It can be seen from Figure 12 that in the upstream of the fire source, the distance of the temperature that spread to the upstream was not long due to the influence of ventilation. When the wind speed was 2.5 m/s, the area with a temperature exceeding 100 °C in the upstream of the tunnel reached 59 m in the longitudinal direction. Upstream of the fire source, the high-temperature areas mainly included the tunnel or relatively high haunch while the temperature of the side wall and roadway was lower. This was because the hot air rose upward and the heat was absorbed by the tunnel vault and the haunch lining. Near the fire source, as the vehicle cross-section and tunnel cross-section were large and due to the influence of ventilation, the temperature of the tunnel vault, haunch, side wall, and roadway was high and there was no obvious stratification phenomenon. Downstream of the fire source, the high-temperate areas also mainly included tunnel or relatively high haunch while the temperature of the side wall and roadway was low. Therefore, from the perspective of rescue, the tunnel fire rescue should start upstream of the fire source. From the perspective of the escape of personnel and vehicles in the tunnel, the personnel and vehicles upstream of the fire source and near the fire source should drive toward the nearest cross aisle immediately after noticing the fire and enter the neighboring tunnel where no fire occurs at full speed. The personnel and vehicles downstream of the fire source should try to escape close to the bottom of the tunnel in order to avoid high-temperature air or inhaling too much smoke.

#### 4.2.3. Longitudinal Temperature Field in the Tunnel Lining

**Figure 13:**
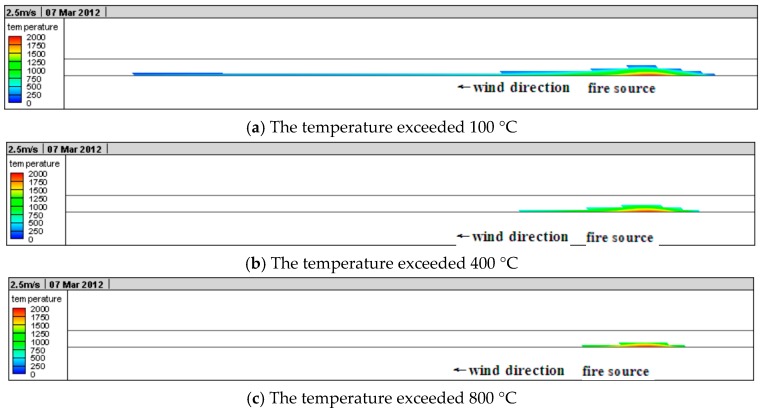
Longitudinal temperature in the tunnel.

The longitudinal temperature variations in the tunnel lining structure are shown in Figure 13.

It can be seen from Figure 13 that the high-temperature zones in the tunnel ling were near the fire source. The influence of fire on the lining in the upstream of the fire source was obviously smaller than on the lining in the downstream. The existing research results have shown that the lining would be influenced to varying degrees when the temperature exceeded 100 °C in the lining. Therefore, we set 100 °C as the boundary to whether the lining structure would be influenced by the fire or not. Figure 13a shows the area with a temperature exceeding 100 °C in the tunnel lining and the total length was 670 m in the longitudinal direction, which was in accordance with the mild damage situation at the site. When the temperature in the lining concrete structure was 400–800 °C, its strength and stability was significantly reduced. The lining concrete structure was exposed to high temperatures for a long time, causing spalling of the lining structure over a wide area. Figure 13b shows the area with a temperature exceeding 400 °C in the tunnel lining and the total length was 300 m. The lining concrete in this area was moderately damaged, which is also in accordance with the moderate damage situation at the site. As the temperature in the lining concrete structure exceeded 800 °C, its strength and stability was dramatically reduced. In this situation, the lining structure experiences cracking, spalling, bursting, and even collapse.

#### 4.2.4. Lateral Temperature Field in the Tunnel Lining

**Figure 14:**
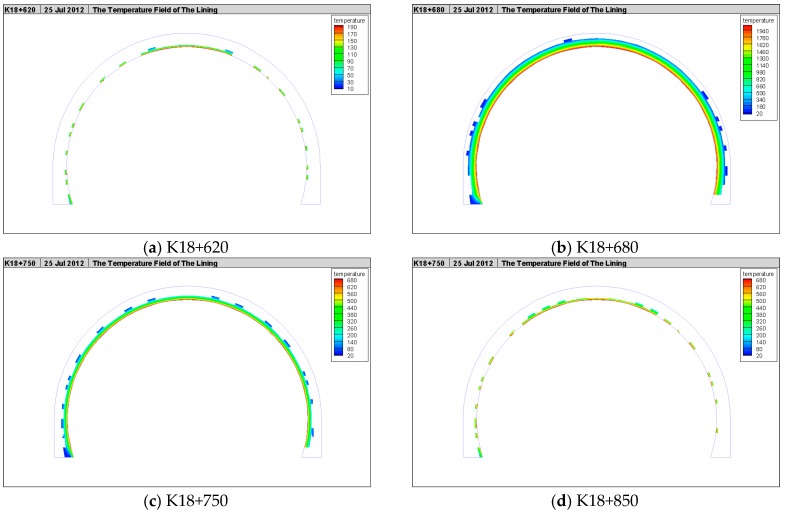
Cross section temperature in the tunnel lining.

The cross-section temperature fields in the tunnel lining at the upstream cross section K18+620, fire source cross-section K18+680, and downstream cross-sections K18+750 and K18+850 are shown in Figure 14.

It can be seen that the vault, haunch, and side wall of the tunnel lining were all influenced by the fire to varying degrees. It can be seen from Figure 14a that the backflow phenomenon of high-temperature air appeared in the upstream of the fire source and the back-flowing hot air and hot smoke mainly gathered at the tunnel vault due to the influence of buoyancy. When the smoke became increasingly denser, the smoke spread to the tunnel haunch and side wall; therefore, the lining at the vault absorbed the most heat energy, followed by the haunch. The last was side wall. The temperature of the vault was the highest, followed by the haunch, and the last was side wall in the tunnel lining. In Figure 14b it can be seen that near the fire source, as the wind speed was not high and the vehicle cross section and the tunnel cross section were relatively large, there was not much difference in the heat radiation energy absorption among the vault, haunch, and side wall at the same cross-section. Besides, due to the influence of ventilation, the air near the fire source was in a turbulent condition and the cross-section was full of hot air; therefore, there was no obvious gradient in the temperature of the vault, haunch, and side wall in the tunnel lining cross section near the fire source. Figure 14c shows that downstream of the fire source, the hot air and hot smoke in the tunnel spread far to the downstream of the fire source due to the influence of ventilation. This cross-section was near the fire source and the air in the tunnel was turbulent. The whole tunnel cross-section was full of smoke and the stratification phenomenon of the temperature of different areas of lining cross section in this section was not obvious. Figure 14d shows that downstream of the fire source (far away from the fire source) the stratification phenomenon of the smoke in the tunnel appeared. The hot air and smoke mainly gathered at the tunnel vault due to the influence of buoyancy, followed by the tunnel haunch and there was a little hot air around the tunnel side wall. The lining at the vault was most influenced, followed by the haunch, and the side wall was least influenced. The thickness with a temperature exceeding 400 °C in the tunnel lining at the fire source was over 30 cm, which was in accordance with the spalling depth of 10–30 cm of in-situ observation.

## 5. Conclusions

In the New Qidaoliang Tunnel, a rear-end collision of two tanker trunks caused a fire. In situ research and laboratory experiments as well as numerical simulation were performed with the aim to evaluate the effect of fire on the tunnel lining structure. The following conclusions can be drawn:

- The fire results in an exfoliation covering an area of 856 m^3^, weakening the solidity of the remaining lining masonry and damaging the road to a length of 650 m. The maximum area loss happened at the spot of the fire with maximum observed concrete spallation up to a thickness of 35.4 cm. The strength of vault and side wall concrete near the fire source was significantly reduced. The loss of concrete strength of side wall near inner surface of tunnel was larger than that near the surrounding rock.
- Numerical simulation indicates that the tunnel went through the process of a small range of area heating up, with an eddy, backflow and lengthway flow happening around the fire during the outbreak of the fire until fire stability. The temperature inside the tunnel changed in accordance with the distance away from the fire, the farther the lower. The downstream of the tunnel was more influential than the upstream. At the same distance away from the fire, the downstream section had a higher temperature than the upstream. Horizontally, the heat mainly gathered in vault or in the higher part—the arch waist for both the upstream and downstream of the fire. The temperature near fire within the tunnel did not show clear stratification.
- The influence of the fire in the upstream is greater than in the downstream. From the perspective of vertical temperature, the length of the damage in all grades is in accordance with the results tested on the spot. The horizontal temperatures within tunnel lining of both downstream and upstream distant from the fire showed a phenomenon of stratification. The highest temperature lies in the vault. There was no clear gradient of temperatures among the vault, the arch waist, and the side wall of transverse section which was near the source of ignition and the downstream part nearby. As for the size of measurement, the thickness where the temperature within tunnel lining near the fire seat was above 800 °C coincides with the depth of exfoliation on field test.
